# Anterior Cruciate Ligament Rupture: Evidence-Based Concepts on Reconstruction, Repair and Future Developments

**DOI:** 10.7759/cureus.67956

**Published:** 2024-08-27

**Authors:** Anthony Thayaparan, Shanjitha Kantharuban

**Affiliations:** 1 Trauma and Orthopaedics, North West Thames Deanery, London, GBR

**Keywords:** orthopaedics trauma, evidence-based, anterior cruciate ligament (acl) reconstruction, acl repair, anterior cruciate ligament (acl)

## Abstract

The anterior cruciate ligament (ACL) is a vital but frequently injured structure. An ACL injury can result in dysfunction, meniscal injuries, and the early onset of osteoarthritis. This article aims to discuss favourable reconstruction techniques through a literature review with consideration for novel methods in order to identify superior methods that provide a patient’s return to function. Current surgical options include reconstruction using different types of autografts and allografts.

## Introduction and background

Anterior cruciate ligament (ACL) tears are commonly seen in the athletic population and may lead to anterior and lateral rotatory instability, requiring surgical reconstruction. Reconstruction techniques vary greatly depending on the choice of graft and technique.

The ACL consists predominantly of type 1 collagen (approximately 90%) and type 3 collagen, with its main blood supply from the middle geniculate artery. It tolerates up to 2200 N of force in the prevention of anterior translation of the tibia on the femur, which provides the majority of stability in this direction of motion. It also contributes to preventing excessive tibial rotation on the femur.

The ACL is typically located, arising from the medial wall of the lateral femoral condyle and typically attaching between the intercondylar eminences of the tibia. Furthermore, the anterior cruciate ligament consists of two bundles with different roles. As described above, the anteromedial bundle typically resists anterior translation of the tibia, while the greatest tension is in knee flexion. The posterolateral bundle, however, is tightest while the knee is in extension and contributes to rotational stability.

A typical presentation of ACL rupture consists of the patient describing a sudden ‘crack’ or ‘pop’ felt deep within the knee, followed by an immediate swelling (or effusion secondary to haemarthrosis). The mechanism varies from direct collision in sports to improper landing or twisting after an action, such as kicking a football or landing after jumping in basketball. Indeed, a clinician must consider other injuries co-existing with an ACL injury. Often, the patient may describe deep-seated pain within the knee, difficulty weight-bearing, and instability, particularly on return to sports such as those that involve rapid changes in direction. Co-existing injuries may consist of a meniscal injury or Segond fracture, an avulsion fracture at the lateral tibia associated with excessive pull from the anterolateral ligament.

Confirmatory tests of an ACL rupture include clinical examinations such as the Lachman’s test and the pivot shift test. The Lachman's test is carried out by flexing the knee 20-30° and translating the tibia forward with an anteriorly directed force. The pivot shift test is carried out with the knee fully extended, placing an internal rotatory and valgus force on the proximal tibia while flexing the knee. If there is a clunk with flexion, this test is positive. Imaging modalities such as plain film radiography may reveal an effusion or fractures associated with this injury. Magnetic resonance imaging is considered the gold standard imaging modality for confirming diagnosis, while computed tomography may have benefits in planning revision surgery.

## Review

While ACL reconstruction has been considered the gold standard for offering superior mechanical stability since the 1990s, there has been a resurgence in attempts to perform ACL repairs. However, more research is required to change the direction of movement from reconstruction to repair. In a case-control study comparing suture-anchor repair (with microfracture) and single-bundle ACL reconstruction, Achtnich et al. purported that the former method was viable for restoring knee stability with comparable functional outcome scores [1]. However, the 15% failure rate in the group with ACL ‘refixation’ is a cause for concern, though the sample sizes in each group were low. Furthermore, this study was performed on those with prior MRI-confirmed proximal avulsion ACL tears, and thus, it cannot be considered a ‘one size fits all’ technique. Table 1 compares both ACL reconstruction and repair.

**Table 1:** Summary of anterior cruciate ligament reconstruction versus repair. ACL: anterior cruciate ligament.

	ACL reconstruction	ACL repair
Advantages	Superior mechanical stability	Proprioception preserved
	Gold standard practice	Avoids donor site morbidity
		Better outcomes during rehabilitation
		Less invasive
Disadvantages	Longer recovery time	Failure
	Osteoarthritic change after surgery	Not suitable for all patients
		Long term data not available

Before expanding into updates in mechanical and biological optimisation, one must consider the current vogue for ACL reconstruction involving the use of bone-patellar tendon-bone (BPTB) grafts or hamstring tendon (HT) grafts (semitendinosus with or without gracilis), with the vast majority of cases with BPTB offering superior joint stability while also associated with more frequent post-operative complications as those identified by Li et al. in a systematic review [2]. Such complications may include anterior knee pain (associated with graft harvest), patella fracture, or tendon rupture, amongst others, though still the more favourable graft choice due to osseous fixation or integration at tibial and femoral tunnels. HT autograft harvest methods involve smaller incisions with tibial tunnel placement in the same incision but may potentially risk iatrogenic injury to saphenous nerve branches and inadequate graft (though this may be rectified by harvest from the contralateral limb). Beyer et al. concluded that overall, BPTB and HT autografts had similar outcomes postoperatively. However, if an HT autograft was used, a double-bundle graft improved function and reduced complications postoperatively [3].

Certainly, one may consider the use of allografts such as fresh frozen patellar tendon (PT), achilles, or hamstring allografts. Interestingly, Siebold et al. found that Achilles tendon allografts were superior to PT grafts. At the same time, Sun et al. performed a randomised controlled trial comparing HT autograft with HT allograft, showing almost identical outcome scores at an average of 7.8 years of follow-up [4,5]. Foster et al. supported this further with autografts showing a mild improvement in failure rate of 4.7 in every 100 versus 8.2 in every 100 allograft reconstructions [6]. While the study was unable to conclude whether any particular allograft was superior to another, they stated that the graft source had a minimal effect on the outcome. Certainly, this shows promise in times of difficulty, such as revision surgery, a lack of appropriate autografts, and multi-ligament reconstruction. While the use of allograft may reduce post-operative donor-site morbidity (such as hamstring weakness or anterior knee pain), it is widely deemed the more expensive option. It relies on the presence of a human tissue bank and coordinating more resources for a steady supply, further adding to costs [7]. This notion is supported by Mistry et al., who state that though non-irradiated allografts are comparable to autografts, they should be considered second-in-line due to their lower cost-effectiveness [8]. They also confirm the paucity of literature around potential graft choices in elite sporting athletes, and one must consider the possible morbidity around autograft harvest and this effect on high-level sport, which is a vital consideration in surgical reconstruction. Similarly, a clinical review carried out by Condello et al. states that allograft tendons are a safe and effective alternative for ACL revision surgery, with comparable failure rates and no increased infection rate when compared to autografts, as long as the tissue is not irradiated [9]. Table 2 compares the main types of grafts used.

**Table 2:** Summary of graft selection BTB: bone-patellar tendon-bone; HT: hamstring tendon [10].

	BTB autograft	HT autograft	Allograft
Advantages	Superior joint stability	Good clinical outcomes	No donor-site morbidity
	Low re-tear rate	Smaller incisions	Failure rate same as autograft over the age of 40
		Low graft failure rate	
Disadvantages	Anterior knee pain	Iatrogenic nerve injury	Higher revision and graft failure rate in young patients
	Long-term osteoarthritic change	Extension loss	
	Risk of a patella fracture	Increased laxity over time	
	Risk of tendon rupture		
	Lower knee extensor strength than HT		

The double-bundle technique, whilst not commonly used as the primary technique of ACL reconstruction, does potentially offer a more anatomical reconstruction and mechanical advantage, mimicking the anteromedial and posterolateral bundles of the native ACL. Unfortunately, the evidence does not favour a particular method (double bundle versus single bundle). Li et al. discuss superior outcomes when assessing pivot-shift or rotational laxity in the double-bundle technique in a meta-analysis, though not conferring any difference in functional recovery and thus no significant difference between the two methods [11]. However, Chen et al. found that at a minimum of five-year follow-up in their meta-analysis, there were no differences in knee stability, clinical function, failure rates, or even rates of osteoarthritic changes [12]. However, this finding was restricted to patients who underwent autologous ACL reconstruction. Suomalainen et al. echo this sentiment but explore the difficulty of the double-bundle technique, which requires two femoral and tibial tunnels each [13]. This would increase operative time and the risk of fracture, and it would pose difficulties in appropriate tunnel placement in such close proximity without causing posterior wall blowout. A recent prospective study by Das et al. demonstrated comparable clinical outcomes between single-bundle versus double-bundle reconstruction. The findings were similar between the groups at one and two years postoperatively [14].

To ensure improvement in anatomical placement of the tunnels, computer-guided single-bundle ACL reconstruction has been thought to improve accuracy. However, this lengthens the operative time and is more invasive, but unfortunately, it does not appear to convey any clinical difference compared to its conventional manual technique counterpart [15]. Endele et al. found no significant differences between computer-assisted and conventionally performed ACL reconstruction in their prospective, randomised controlled study of 40 patients at two years, comparing tunnel placement and clinical outcomes [16]. Zhu et al. used the idea of computer-navigated ACL reconstruction and compared single bundle with double bundle reconstruction [17]. After a minimum of two years of follow-up, they found no significant difference in clinical outcomes between the two studied groups in their retrospective study of 42 patients, but they stated that this method may prevent the misplacement of necessary tunnels by less experienced surgeons.

Furthermore, 3D printing technology has given notable outcomes and provides tailored solutions to unique clinical challenges by utilising patient-specific imaging data [18]. A recent cadaveric study by Zee et al. demonstrated femoral tunnel positioning to be augmented in accuracy and consistency by using patient-specific 3D-printed surgical guides [19]. Chen et al. demonstrated in their recent study that using 3D printing technology allows for high reliability and reproducibility in femoral bone tunnel preparation [18].

Further developments in aid of mechanical stability in ACL surgery include that which augments ACL repair rather than reconstruction due to the far higher incidences of failure in repair. One such method that shows promising results is internal brace ligament augmentation (IBLA). Mackay et al. studied 68 patients who underwent primary ACL repair with the placement of a 2.5-mm polyethylene tape passed from the tibia, through the ACL, into the femur and secured [20]. Whilst this study included only a minimum of one-year follow-up, the results were promising despite the cohort being those with only femoral-sided avulsions. One patient required revision surgery due to failure on return to ‘collision’ sports at 18 weeks, whilst three others required surgery for meniscal pathology, arthrofibrosis, and chondral pathology each. A recent systematic review demonstrated that HT, quadriceps tendon and BPTB grafts augmented with high-strength suture tape (internal bracing) are effective methods for ACL reconstruction [21]. This review showed significant biomechanical or clinical advantages compared to standard ACL reconstruction.

Similarly, dynamic intraligamentary stabilisation (DIS) aims to stabilise the repaired ACL with a secured preloaded spring within the tibia that provides a constant posterior force on the tibia, attached to which is a 1.8 mm polyethylene braided tape that again traverses the knee joint through the repaired ACL, secured with a button on the femoral side, and extensive microfracturing performed at the side of the avulsion to stimulate healing (not too dissimilar from the IBLA). Bieri et al. did find that with this technique, patients returned to work one month earlier than their matched ACL reconstructed counterparts, but added that those treated with DIS were repaired acutely with a mean time from injury to fixation of 14 days (with the recommendation to be carried out within three weeks) in comparison with the reconstruction cohort [22]. Häberli et al., however, found that while 8.7% of the 455 patients undergoing ACL repair and DIS required revision surgery (ACL reconstruction), a significantly larger proportion of patients (almost 40%) required other re-interventions such as removal of hardware, arthroscopy for scar tissue debridement, and treatment of meniscal pathology, as well as manipulation under anaesthesia [23]. Similarly, a more recent prospective study by Endreß et al. demonstrated a significantly greater range of motion deficits and revision rates after DIS compared to their ACL reconstruction group [24].

Mechanical stability has been the main aim in ACL reconstruction and repair until recently, when biological advances in medicine have helped augment surgical technique. Mahapatra et al. discuss recent findings such as the lack of effectivity in improving biomechanical ACL strength when augmenting repair with engineered collagen bio-scaffolds or platelet-rich plasma (PRP) supplementation (despite the latter increasing collagen gene expression in fibroblasts) [25]. However, in combination, it is thought that the collagen bio-scaffold may enhance the effects of PRP in stimulating healing in ACL defects, leading to a 40% improvement in ligament strength at six weeks compared to no treatment [26]. As a follow-up, the same group found that in Yorkshire pigs, the same technique improved ACL repair on MRI at four weeks compared to isolated ACL repair in the contralateral knee (with no biological enhancement). Furthermore, the collagen-PRP hydrogel used improved the biomechanical properties of the repaired ACLs [27].

Possibly the most promising work of the same primary author recently published showed that a bio-enhanced ACL repair was comparable to bio-enhanced ACL reconstruction and traditional ACL reconstruction in Yucatan minipigs, but more importantly, offered a level of chondral protection (reducing post-traumatic arthritis) following surgery [28]. This specific type of repair consists of placing a resorbable protein-based implant between the torn ends of the ACL and injecting autologous blood within it. Furthermore, the bridge-enhanced ACL repair (BEAR), spawned from the aforementioned bio-enhanced repair, found similar outcomes in anteroposterior laxity and patient-reported outcome measures when compared with ACL reconstruction in a prospective randomised controlled trial at two years [29]. With these extremely encouraging results, one must, however, consider the mechanical augmentation to the torn ACL remnants in addition to the BEAR, similar to the aforementioned IBLA but also the younger patient cohort in this study with a median age of 17 years (favourable physiology conducive to healing).

In the case of the preservation of the ACL remnant, there is still much contention. It may have a role in improving the vascularisation, ligamentisation and reinnervation of grafts. However, this has still not been proven clinically and may lead to the risk of impingement and the development of a cyclops lesion [30]. The combination of reconstruction and retaining the ACL remnant leads to innovations such as the BEAR, or single anteromedial bundle biological augmentation (SAMBBA) technique, which involves retaining the ACL stump and tunnelling the single anteromedial bundle of the semitendinosus while retaining the hamstring distal insertion [31].

## Conclusions

In addition to the various techniques discussed in this article, one must consider other factors of ACL reconstruction, such as optimal tunnel placement, techniques such as transtibial or ‘all-inside’ approaches, and the consideration of retaining an ACL remnant, amongst others. Indeed, with the rise of various new techniques and variations to existing techniques, it is, without doubt, integral to consider mechanical and biological optimisation in ACL reconstruction and, perhaps, in the future, repair. Further studies, ideally comparing the best of each, are required to identify new gold standards or confirm existing ones. By fusing ideas in our understanding of biological and mechanical enhancement of ACL reconstruction and repair, we will find which is deemed the best for return to function and stability with the greatest patient-reported outcome measures.
